# Evaluation of major salivary glands with ultrasonography in multiple sclerosis patients

**DOI:** 10.1186/s12903-024-04007-0

**Published:** 2024-02-16

**Authors:** Mustafa Kıranatlı, Melike Yurttaş, Müjgan Güngör, Sibel Canbaz Kabay

**Affiliations:** 1 Faculty of Dentistry, Oral and Maxillofacial Radiology, Afyon Health Sciences University, Afyon, Turkey; 2 Faculty of Dentistry, Oral and Maxillofacial Radiology, Kütahya Health Sciences University, Kütahya, Turkey; 3 Faculty of Dentistry, Oral and Maxillofacial Radiology, Lokman Hekim University, Ankara, Turkey; 4 Faculty of Medicine, Neurology Department, Dokuz Eylul University, İzmir, Turkey

**Keywords:** Multiple sclerosis, Ultrasonography, Salivary glands

## Abstract

**Background:**

This study aimed to investigate the homogeneity of the major salivary glands in multiple sclerosis (MS) patients using ultrasonography (USG), assess DMFT indices, measure resting salivary flow rates, and compare these values with healthy individuals.

**Methods:**

In this study, 20 individuals diagnosed with Relapsing-Remitting Multiple Sclerosis (RRMS) (mean age 36.15 ± 8.51) and 20 systemically healthy individuals (mean age 35.7 ± 9.22) were included. Oral and radiologic examinations were performed in all individuals. The parotid and submandibular salivary glands were examined using USG, and their homogeneity was assessed based on the scoring system recommended by the Outcome Measures in Rheumatology Clinical Trials (OMERACT) study group. Resting salivary flow rates and DMFT indices were measured, and the obtained data were subjected to statistical analysis.

**Results:**

The parotid glands exhibited more heterogeneity on USG within the MS patient group than the control group, with a statistically significant difference between the two groups (*p* = 0.015). A statistically significant correlation was observed in total homogeneity values between the patient and control groups (*p* = 0.021). Furthermore, the MS patient group exhibited higher DMFT indices and lower salivary flow rates than the control group. The difference between the DMFT indices and salivary flow rate between the patient groups was statistically significant (*p* = 0.004 and *p* = 0.002 respectively).

**Conclusions:**

The parenchyma of the major salivary glands in MS patients exhibited decreased homogeneity than the healthy controls. Additionally, the MS group showed a decrease in salivary flow rate and an increase in the DMFT index. Autonomic dysfunction and medications used for MS are thought to cause salivary gland hypofunction and dry mouth. It can be interpreted that hyposalivation and motor skill losses in MS patients lead to an increase in DMFT index.

## Introduction

Multiple sclerosis (MS) is a chronic, autoimmune disease of the central nervous system, characterized by inflammation, demyelination and axonal damage [1]. MS usually starts in young adults, between the ages of 20 and 40 and women are affected approximately three times more [2]. There are different subtypes of MS as relapsing-remitting MS (RRMS), primary-progressive MS and secondary progressive MS. These patients generally have complaints such as vision problems, fatigue, motor weakness, cognitive and coordination disorders. The “Expanded Disability Status Scale” (EDSS) is widely used in the evaluation of disability in MS patients. In this scale, MS is evaluated between 0 and 10, where normal neurological examination is 0 and MS-related death is 10 [3].

Xerostomia is defined as the subjective complaint of dry mouth, which can impact speech, chewing, swallowing, denture-wearing, and overall well-being. Subjective oral findings such as dry mouth sensation, dysphagia, taste changes, tongue numbness, burning sensation and halitosis have been reported from MS patients in the studies [4, 5]. Mortazavi et al. detected that the amount of salivary flow was lower in MS patients than the control group [6]. In a study about MS and dental caries with the DMFT index, researchers found the DMFT index to be significantly higher in individuals with MS compared to the control group [7].

In dentistry, ultrasonography (USG) is employed to assess various aspects, including the salivary glands, chewing muscles, orofacial swellings, lymph nodes, etc. The interest in USG has grown due to its non-ionizing radiation, non-invasiveness, and ease of application in daily clinical practice [8, 9]. In USG, the normal anatomy of salivary glands typically exhibits homogeneous echogenicity. In inflammatory diseases, cystic and tumoral conditions can lead to changes in the echogenicity of the glands in USG. In such cases, one may observe gland enlargement and the presence of single or multiple hypoechoic or anechoic nodules [10].

While dry mouth has been identified in assessments of MS patients, no studies have investigated the evaluation of salivary glands in MS patients using USG. This study aimed to examine possible salivary gland changes in MS patients using USG. Additionally, the DMFT index and salivary flow rate of MS patients were evaluated, and all data obtained from the patients were compared with those of healthy individuals.

## Methods

This study was carried out in Kütahya Health Sciences University Faculty of Dentistry, Department of Oral and Maxillofacial Radiology and Neurology Department of Faculty of Medicine. The study was approved by the Kütahya Health Sciences University Clinical Research Ethics Committee with the decision dated 16.11.2021 and numbered 2021-07/03. Informed consent was obtained from the individuals participating in the study in accordance with the Helsinki Declaration decisions.

### Patient selection

This study included 20 MS patients and 20 age- and sex-matched healthy controls. The MS patient group was selected from the patients in the Neurology Department of the university and while the control group consisted of healthy individuals who applied various dental complaints to the Oral and Maxillofacial Radiology Department.

The inclusion criteria were as follows: participants had to be over the age of 18, have no systemic disease other than MS (RRMS) that might affect salivation, not using a drug known to cause dry mouth in MS treatment (glatiramer acetate). Additionally participiants needed to have experienced their last MS attack 6 months ago, exhibit low disability levels (EDSS ≤3.5), and not have conditions that could mimic dry mouth symptoms, such as mouth breathing. The control group comprised individuals without any systemic disease, no medical history related to the salivary glands (including surgery, pathology, chronic diseases, and drug use), and was matched the patient group in terms of age and gender. The study excluded MS patients with frequent attacks, those under the age of 18, individuals using medications known to reduce saliva production, those with another systemic disease in addition to MS, and those with a high level of disability (EDSS > 3.5). Participants unable to provide consent due to conditions such as mental retardation or illiteracy, those with a medical history related to salivary glands, and individuals using any drugs that might affect salivation were excluded from the study.

### Measurement of saliva flow rate

The salivary flow rate was evaluated by measuring unstimulated salivary flow. Patients did not eat or drink anything for at least 1 hour before taking the measurement. Following a request for patients to rinse their mouths with 10 ml of distilled water for 30 seconds, they were then asked to collect unstimulated saliva into millimeter-marked distilled tubes. The procedure involved positioning the individuals’ heads forward for 10 minutes without spitting. They were instructed to let saliva flow naturally from their lips into the collection tube. The unstimulated salivary flow rate was calculated in milliliters/minute by dividing the obtained measurement by the time.

### Evaluation with ultrasonography

The right-left submandibular and parotid salivary glands of all patients were examined using a 13 MHz linear probe with USG device (Sonosite M-Turbo FUJIFILM Sonosite, Inc. WA, USA). The examinations were conducted by an Oral and Maxillofacial Radiologist (M.K.) with 4 years of experience. During the USG examination, the patient’s head was fixed to the headrest in order to minimize mobility and ensure standardization. In all patients, the depth of the examined region was set to 4 cm on the device. During the examination of both salivary glands, the patient’s head was turned to face the opposite direction of the examined region.

### Evaluation of the homogeneity of the salivary gland

The scoring system recommended by the Outcome Measures in Rheumatology Clinical Trials (OMERACT) study group was used to evaluate the parenchymal homogeneity. The OMERACT working group developed a four-grade semi-quantitative USG scoring system based on parenchymal homogeneity in Sjogren Syndrome patients. According to this scoring system;

Grade 0; normal parenchyma.

Grade 1; minimal change: slight inhomogeneity without anechoic/hypoechoic areas.

Grade 2, moderate variation: moderate inhomogeneity with focal anechoic/hypoechoic areas.

Grade 3; severe change: diffuse heterogeneity with anechoic/hypoechoic areas covering the entire gland surface [11].

### DMFT index

Detailed intraoral examinations of all individuals of the teeth were dried and performed under reflector light with a probe and a mirror. Existing panoramic and digital bite-wing radiographs of all participants were examined carefully. The DMFT index was obtained by summing the number of caries (D), extracted (M), and filled teeth (F). Congenitally missing, unerupted, or supernumerary teeth were not included.

### Statistical analysis

Analyzes were performed in IBM SPSS® Statistics for Windows® 25.0 (IBM®, Chicago, IL, USA). As the first step of the statistical analysis, the assumption of normality was checked with the Shapiro-Wilk test. Independent Sample T-test was used to compare the means of two independent groups with normal distribution. Mann Whitney U test was used to compare the means of two independent groups that did not have a normal distribution. ANOVA test was performed to compare the means of three or more independent groups with normal distribution. The Post Hoc Bonferroni test was applied to reveal the group or groups that made the difference. Kendal’s Tau correlation was used to measure the relationship between the ordinal categorical variable and continuous variables. In the analysis of categorical data, Fisher’s Exact test was applied when the sample size was not sufficient (*n* < 5). *p*-values < 0.05 were considered statistically significant.

## Results

A total of 40 patients, 20 MS patients without any other systemic disease (mean age 36.15 ± 8.51) and 20 healthy (mean age 35.7 ± 9.22 years) individuals, were included in this study. The patients were divided into 3 groups according to their age (20-30 years old, 31-41 years old, 42 years old and above). Using their EDSS scores, the MS group was separated into two groups: 1-1.5 (*n* = 12) and 2-3.5 (*n* = 8).

The homogeneity levels of salivary glands were lower than in the control group. While the difference in parotid gland ultrasonography (USG) homogeneity between the MS patients and control groups was statistically significant (*p* = 0.015), no statistically significant correlation was observed between the groups regarding submandibular gland homogeneity. (*p* = 0.202) A statistically significant relationship was observed between the MS and control groups concerning the homogeneity of the left parotid, right submandibular gland, and total salivary glands. (*p* = 0.005, *p* = 0.004, *p* = 0.021 respectively) (Table 1).

**Table 1:** Relation between Patient Groups and USG Homogeneities

	USG Scores	Study group		*p*
		Patient group (n %)	Control group (n %)	
USG homogeneity of the right parotid gland	0	10 (50)	15 (75)	.196
	1	9 (45)	5 (25)	
	2	1 (5)	0	
USG homogeneity of the left parotid gland	0	7 (35)	17 (85)	.005*
	1	11 (55)	3 (15)	
	2	2 (10)	0	
USG homogeneity of right submandibular gland	0	1 (5)	3 (15)	.004*
	1	11 (55)	17 (85)	
	2	7 (35)	0	
	3	1 (5)	0	
USG homogeneity of left submandibular gland	0	2 (10)	2 (10)	.666
	1	10 (50)	13 (65)	
	2	8 (40)	5 (25)	
Total salivary gland USG homogeneity	0	0	1 (5)	.021*
	1	2 (10)	2 (10)	
	2	1 (5)	7 (35)	
	3	5 (25)	8 (40)	
	4	4 (20)	2 (10)	
	5	4 (20)	0	
	6	2 (10)	0	
	7	2 (10)	0	

*p < 0.05 (Fisher’s Exact)

DMFT indices were higher and the mean salivary flow rate was lower in the MS group. The difference between the DMFT and salivary flow rate means was statistically significant. (*p* = 0.004, *p* = 0.002 respevticely) (Table 2).

**Table 2:** Comparison of the Means of DMFT and Salivary Flow Rates with Patient Groups

	Study group	Mean	*p*
DMFT	Patient group	8.50 ± 4.63	0.004*
	Control group	4.75 ± 2.76	
Salivary Flow Rates	Patient group	0.27 ± 0.05	0.002*
	Control group	0.33 ± 0.05	

*p < 0.05 (Independent Sample T-test) (Mann Whitney U)

The patient group with EDSS scores of 1-1.5 exhibited lower DMFT means than the group with scores of 2-3.5, and they also demonstrated higher salivary flow rates. While the salivary flow rate showed a significant difference (*p* = 0.002) no statistically significant difference in DMFT indices between the EDSS groups (*p* = 0.339) (Table 3).

**Table 3:** Comparison of DMFT and Salivary Flow Rate Means According to EDSS Scores in the Patient Group

	EDSS	*n*	Mean	*p*
DMFT	0-1.5	12	7.66 ± 4.55	.339
	2-3.5	8	9.75 ± 4.77	
Salivary Flow Rates	0-1.5	12	0.29 ± 0.05	.002*
	2-3.5	8	0.25 ± 0.04	

*p < 0.05 (Independent Sample T-test)

In the MS group, a statistically significant, negative, and moderate correlation (correlation coefficient: − 0.367) was observed between submandibular gland USG homogeneity and salivary flow rate. In the MS group, a statistically significant, negative, and moderate correlation was found between the DMFT score and salivary flow rate. (correlation coefficient: − 0.475) In the control group, the correlation coefficient between the DMFT score and salivary flow rate was − 0.430, and it was also statistically significant, negative, and moderate (Table 4).

**Table 4:** The Correlation Between DMFT and Salivary Flow Rate Measurements and Ultrasound Scores of Participants

		Patient Group		Control Group	
		DMFT	Salivary Flow Rates	DMFT	Salivary Flow Rates
USG homogeneity of parotid gland	Rho	−.032	−.082	.100	−.031
	p	.862	.652	.608	.874
USG homogeneity of submandibular gland	Rho	.154	−.367	.034	.000
	p	.393	.041*	,855	1.000
Total salivary gland USG homogeneity	Rho	.088	−.312	,115	−.064
	p	.617	.072	,531	.727
DMFT	Rho		−.475		−.430
	p		.005*		.013*

*p < 0.05 (Kendal’s Tau)

## Discussion

MS is a disease that can occur at any age but typically affects young adults. The first symptoms usually emerge between the ages of 20 and 50 [12]. Studies on MS report a mean age in patient groups ranging between 36 and 50 years [4, 12–14]. In alignment with the literature, the mean age of the 20 MS patients included in this study was 36.15 ± 8.51 years. MS affects females more than males [2]. It is believed that multiple factors, including gonadal hormones, differences in the immune or nervous systems between women and men, genetic predispositions, various environmental exposures, and the effects of modern lifestyle, may contribute to the higher occurrence of MS in women [13]. Also, the number of female patients was higher in this study. (female/male ratio 1.85:1).

Inflammatory or neoplastic conditions can cause enlargement of the salivary glands, while sclerosing diseases may lead to gland atrophy [14]. Additionally, systemic diseases such as diabetes and hypertension can potentially impact the size and function of the salivary glands [15]. For this reason, in this study, the patient group was selected from individuals with only MS who do not have any other systemic disease to prevent any potential impact on the submandibular and parotid glands from other pathologies, and the control group was comprised of healthy individuals.

Saliva is frequently employed in disease diagnosis due to its easy accessibility and non-invasiveness. It can be collected without the need for any equipment and has the capacity to reflect the inflammatory status of the entire mouth. Mortazavi et al. [6] reported a significant reduction in saliva flow rate among MS patients compared to healthy controls. In this study, a statistically significant difference was found in the unstimulated saliva flow rate between the two groups.

There have been reports indicating that salivary gland USG scores are correlated with both clinical and serological findings in Sjogren’s syndrome. In studies comparing the homogeneity of salivary glands in individuals with Sjogren’s syndrome and a healthy control group using USG, the scores of patients with Sjogren’s syndrome were significantly higher than those of the control group [16–19]. Lee et al. [20] reported that the salivary gland parenchyma of patients with systemic sclerosis exhibits greater heterogeneity than in individuals with idiopathic sicca syndrome and less heterogeneity than in individuals with primary Sjögren’s syndrome. Badarinza et al. [21] evaluated the parotid and submandibular salivary glands of obese and diabetic patients and healthy individuals using USG. Regarding the parotid and submandibular glands’ elasticity modulus, homogeneity, and echogenicity, they found no variations between the groups concerning age or gender. Hora et al. [22] reported that 43.75% MS patients had parotid with heterogeneous echotexture. In this study, the homogeneity levels of salivary glands were lower than in the control group and four (%20) MS patients stated that they felt dry mouth at different times during the day. Studies have found that MS patients exhibit symptoms of Sjögren’s syndrome, such as xerostomia and xerophthalmia [22, 23]. It is suggested that the pathogenic mechanism underlying xerophthalmia and xerostomia in MS differs from that known in Sjögren’s syndrome. The association between sicca complex symptoms and MS patients is believed to be related to autonomic dysfunction in MS [23].

The EDSS is a widely used scale to assess the degree of disability of MS patients. The increase in EDSS in MS patients may also affect oral hygiene practices with reduced dexterity. Patients with an EDSS score of 3.5 and lower were included in this study due to increased disability, bedriddenness, deterioration in functional systems, and multiple drug use. Dulamea et al. [24] reported that there was no significant relationship between EDSS score and periodontal parameters. Hatipoğlu et al. [4] found that the plaque index, gingival index, and probing depth values were higher in the group with EDSS > 3.5 compared to the group with EDSS ≤3.5, and the clinical attachment loss values were similar between the two groups. In this study, there was no statistically significant difference in DMFT and USG homogeneity scores between the EDSS groups, a significant difference was observed in saliva flow rate.

It has been reported that MS patients exhibit moderate to severe plaque accumulation, and their oral hygiene is lower than the national averages [25, 26]. The DMFT index is used to determine how much a community is affected by tooth decay and its consequences. The findings of certain studies investigating the relationship between MS and oral health with the DMFT index are contradictory. In the study conducted by McGrother et al. [7] the DMFT index of MS patients was significantly higher compared to the control group. Kovac et al. [27] stated that the number of missing teeth in MS patients was significantly higher than in the control group. In the other two studies comparing MS patients and control groups, no statistically significant difference was observed in terms of the DMFT index [26, 28]. In this study, the DMFT index of MS patients was found to be significantly higher than the control group. Although the DMFT index of patients with an EDSS score between 0 and 1.5 was lower than that of those between 2 and 3.5. (*p* > 0.005) According to these results, it can be thought that the risk of caries is higher in MS patients than in the healthy population. Due to the increase in MS duration and a high degree of EDSS, the inability of patients to perform oral care effectively has led to an increase in the DMFT index, which may have increased the number of caries, filled teeth, and tooth loss.

In this study, 20 people with MS were included in the study. The low number of patients may be one of the limitations of our study. Studies with a larger number of patients may give more accurate results in terms of generalizing the data. Another limitation is that the healthy individuals participating in the study are selected only on the basis of anamnesis without requesting any additional medical screening. It should also be taken into account that there may be individuals who are unaware of their disease and have not yet been diagnosed.

## Conclusion

The present study found that loss of homogeneity was observed in the submandibular and parotid glands of MS patients compared to the healthy control group. Additionally, resting saliva flow was found to be lower in MS patients than in the healthy control group. The higher DMFT index in MS patients compared to healthy controls suggests an increased risk of caries and associated consequences. This elevated risk may be attributed to factors such as a decrease in saliva flow rate, oral hygiene practices, nutritional habits, socio-economic conditions, and motor skill losses.

## Data Availability

No datasets were generated or analysed during the current study.

## Abbreviations

MS: Multiple Sclerosis
RRMS: Relapsing-Remitting Multiple Sclerosis
USG: Ultrasonography
EDSS: Expanded Disability Status Scale

## References

[1] Oh J, Vidal-Jordana A, Montalban X (2018). Multiple sclerosis: clinical aspects. Curr Opin Neurol.

[2] Greer JM, McCombe PA (2011). Role of gender in multiple sclerosis: clinical effects and potential molecular mechanisms. J Neuroimmunol.

[3] Kurtzke JF (1983). Rating neurologic impairment in multiple sclerosis: an expanded disability status scale (EDSS). Neurology.

[4] Hatipoglu H, Kabay SC, Hatipoglu MG, Ozden H (2016). Expanded disability status scale-based disability and dental-periodontal conditions in patients with multiple sclerosis. Med Princ Pract.

[5] Villa A, Connell CL, Abati S (2014). Diagnosis and management of xerostomia and hyposalivation. Ther Clin Risk Manag.

[6] Mortazavi H, Akbari M, Sahraian MA, Jahromi AA, Shafiei S (2020). Salivary profile and dental status of patients with multiple sclerosis. Dent Med Probl.

[7] McGrother C, Dugmore C, Phillips M, Raymond N, Garrick P, Baird W (1999). Multiple sclerosis, dental caries and fillings: a case-control study. British Dent J.

[8] Carotti M, Salaffi F, Di Carlo M, Barile A, Giovagnoni A (2019). Diagnostic value of major salivary gland ultrasonography in primary Sjögren’s syndrome: the role of grey-scale and colour/power Doppler sonography. Gland Surg.

[9] Orhan K. Introduction to ultrasonography in Dentomaxillofacial imaging. In: Kaan O, editor. Ultrasonography in Dentomaxillofacial Diagnostics. Switzerland, Springer; 2021. p. 1–5.

[10] Delantoni A, Kaan O (2021). Sonographic Anatomy and Pathology Salivary Glands. Ultrasonography in Dentomaxillofacial Diagnostics.

[11] Huang PH, Chen DY (2021). Diagnostic value of the salivary gland ultrasonography scoring system in patients with primary Sjogren's syndrome. J Med Ultrasound.

[12] Compston A, Coles A (2008). Multiple sclerosis. Lancet.

[13] Harbo HF, Gold R, Tintoré M (2013). Sex and gender issues in multiple sclerosis. Ther Adv Neurol Disord.

[14] Dodds MW, Johnson DA, Yeh C-K (2005). Health benefits of saliva: a review. J Dent.

[15] Mata AD, Marques D, Rocha S, Francisco H, Santos C, Mesquita MF (2004). Effects of diabetes mellitus on salivary secretion and its composition in the human. Mol Cell Biochem.

[16] Qi X, Sun C, Tian Y, Han Y, Peng C, Jin H (2017). Comparison of the diagnostic value of four scoring systems in primary sjögren's syndrome patients. Immunol Lett.

[17] Zhang X, Feng R, Zhao J, Wang Y, He J, Liu L (2021). Salivary gland ultrasonography in primary Sjögren's syndrome from diagnosis to clinical stratification: a multicentre study. Arthritis Res Ther.

[18] Zhang X, Zhang S, He J, Hu F, Liu H, Li J (2015). Ultrasonographic evaluation of major salivary glands in primary Sjögren's syndrome: comparison of two scoring systems. Rheumatology (Oxford).

[19] Lee K-A, Lee S-H, Kim H-R (2018). Diagnostic and predictive evaluation using salivary gland ultrasonography in primary Sjögren’s syndrome. Clin Exp Rheumatol.

[20] Lee KA, Choi W, Kim J, Kim HS (2021). High prevalence of salivary gland ultrasound abnormalities in systemic sclerosis. Joint Bone Spine.

[21] Badarinza M, Serban O, Maghear L, Bocsa C, Micu M, Porojan MD (2019). Multimodal ultrasound investigation (grey scale, Doppler and 2D-SWE) of salivary and lacrimal glands in healthy people and patients with diabetes mellitus and/or obesity, with or without sialosis. Med Ultrason.

[22] Hora JSI, da Silva MCR, Braga CLS, Loureiro AM, Alves ATNN, Lourenço SQC (2022). Dry oral and ocular manifestations and autoantibodies characteristic of primary Sjögren's syndrome in multiple sclerosis. Mult Scler Relat Disord.

[23] Masi G, Annunziata P (2016). Sjögren's syndrome and multiple sclerosis: two sides of the same coin?. Autoimmun Rev.

[24] Dulamea AO, Boscaiu V, Sava MM (2015). Disability status and dental pathology in multiple sclerosis patients. Mult Scler Relat Disord.

[25] Manconi B, Liori B, Cabras T, Vincenzoni F, Iavarone F, Lorefice L (2018). Top-down proteomic profiling of human saliva in multiple sclerosis patients. J Proteome.

[26] Symons AL, Bortolanza M, Godden S, Seymour G (1993). A preliminary study into the dental health status of multiple sclerosis patients. Spec Care Dentist.

[27] Kovač Z, Uhač I, Buković D, Ćabov T, Kovačević D, Gržić R (2005). Oral health status and temporomandibular disorders in multiple sclerosis patients. Coll Antropol.

[28] Tavanga A, Etemadifar M, Emamjomeh M, Mojtahedi N. Dental amalgam and multiple sclerosis: a case-control study. Oral Maxillofac Pathol J. 2018;9(1)

